# Genome Sequence of Staphylococcus aureus Strain CBS2016-05, Isolated from Contaminated Platelet Concentrates in Canada

**DOI:** 10.1128/MRA.00288-21

**Published:** 2021-08-26

**Authors:** Basit Yousuf, Annika Flint, Kelly Weedmark, Franco Pagotto, Sandra Ramirez-Arcos

**Affiliations:** a Centre for Innovation, Canadian Blood Services, Ottawa, Canada; b Department of Biochemistry, Microbiology, and Immunology, University of Ottawa, Ottawa, Canada; c Bureau of Microbial Hazards, Health Products and Food Branch, Health Canada, Ottawa, Canada; University of Maryland School of Medicine

## Abstract

We present the genome sequence of Staphylococcus aureus strain CBS2016-05, which was isolated from contaminated platelet concentrates by Canadian Blood Services in 2016. This strain caused a septic reaction in an acute leukemia patient. Genome sequence analysis revealed the presence of one chromosome (2,766,936 bp) and one plasmid (36,441 bp).

## ANNOUNCEMENT

Staphylococcus aureus is an opportunistic pathogen that occurs ubiquitously in the upper respiratory tract and can colonize human skin (1). S. aureus is a recurrently reported bacterial contaminant of platelet concentrates (PCs), a therapeutic blood component that is used to treat patients with deficient platelet counts. S. aureus has emerged as a safety threat to transfusion patients in recent years because it occasionally escapes detection during PC screening with the automated BACT/ALERT culture system (2). Here, we present the genome of S. aureus strain CBS2016-05, which was isolated from contaminated PCs and implicated in a septic transfusion reaction in an acute myeloid leukemia patient (3) as part of a hemovigilance investigation of bacterial contamination in PCs in Canada.

S. aureus CBS2016-05 was isolated from patient samples and residual PCs obtained after the transfusion event. Upon isolation and identification, bacterial suspensions were prepared in brain heart infusion medium supplemented with 15% glycerol and stored frozen at −80°C. For DNA isolation, CBS2016-05 was streaked onto blood agar plates, and single colonies were cultured at 35°C overnight in 5 ml Trypticase soy broth with 0.6% yeast extract (4). Cells were collected by centrifugation and resuspended in DNA/RNA Shield reagent (Cedarlane). DNA extraction was performed with the Zymo Quick-DNA HMW MagBead kit (Cedarlane) with lysozyme and RNase A treatment according to the manufacturer’s instructions (Zymo Research Corp.).

Illumina paired-end (2 × 300-bp) whole-genome shotgun (WGS) data were generated using Nextera XT libraries run on a MiSeq instrument (v3 chemistry) according to the manufacturer’s protocol (Illumina). Illumina reads (*n* = 1,826,796 reads) were processed using fastp v0.20.0 (5) to remove adapter and barcode sequences, to correct mismatched bases in overlaps, and to filter low-quality reads. Nanopore WGS data (*n* = 29,813 reads) were obtained using the rapid barcoding sequencing kit (SQK-RBK004) and 1D MinION chemistry (R9.4 FLO-MIN106 flow cell) according to the manufacturer’s protocol (Oxford Nanopore Technologies) (6). Long-read processing was performed using Guppy GPU v3.3.3+fa743ab, and reads of <1 kb were removed using Filtlong v0.2.0 (https://github.com/rrwick/Filtlong). A *de novo* assembly (Unicycler v0.4.8, normal mode, default circularization and rotation) (7) was generated using filtered Illumina (1,816,530 reads, with 162× coverage) and Nanopore (23,007 reads [*N*_50_ value of 15,180 bp], with 78.4× coverage) reads. Annotation was performed using Prokaryotic Genome Annotation Pipeline (PGAP) v2020-09-24.build4894 (best-placed reference protein set, GeneMarkS-2+) (https://github.com/ncbi/pgap), and metrices were analyzed with QUAST v5.0.2 (https://github.com/ablab/quast) (8). For all computational tools, default parameters were used except where otherwise noted.

The closed S. aureus CBS2016-05 genome (GC content of 32.8%) comprises a 2,766,936-bp chromosome and a 36,441-bp plasmid. A total of 2,603 genes, 128 pseudogenes, 0 CRISPR loci, 19 rRNAs, 59 tRNAs, and 4 noncoding RNAs were identified. S. aureus CBS2016-05 was assigned to sequence type 182 (ST182) based on the PubMLST database (9).

### Data availability.

This WGS project is available in GenBank and the Sequence Read Archive (SRA) under the accession numbers listed in Table 1.

**TABLE 1:** Provenance, sequencing, and assembly of the CBS2016-05 isolate

Parameter	Finding
Isolate name	CBS2016-05
BioProject accession no.	PRJNA703931
GenBank accession no.	
Chromosome	CP070991
Plasmid	CP070992
SRA accession no.	
Illumina reads	SRR13745245
Nanopore reads	SRR13745251
Location of isolation	Toronto, Canada
Year of isolation	2016
